# Single cell isolation process with laser induced forward transfer

**DOI:** 10.1186/s13036-016-0045-0

**Published:** 2017-01-13

**Authors:** Yu Deng, Philippe Renaud, Zhongning Guo, Zhigang Huang, Ying Chen

**Affiliations:** 1 School of Electromechanical Engineering, Guangdong University of Technology, Guangzhou, 510006 China; 2 Microsystems Laboratory, STI-LMIS, Ecole Polytechnique Federale de Lausanne, CH-1015 Lausanne, Switzerland; 3 Guangdong Provincial Key Laboratory of Functional Soft Condensed Matter, Guangzhou, 510006 China

**Keywords:** Single cell isolation, Laser induced forward transfer, Proliferation, Mechanical stress, Cell damage

## Abstract

**Background:**

A viable single cell is crucial for studies of single cell biology. In this paper, laser-induced forward transfer (LIFT) was used to isolate individual cell with a closed chamber designed to avoid contamination and maintain humidity. Hela cells were used to study the impact of laser pulse energy, laser spot size, sacrificed layer thickness and working distance. The size distribution, number and proliferation ratio of separated cells were statistically evaluated. Glycerol was used to increase the viscosity of the medium and alginate were introduced to soften the landing process.

**Results:**

The role of laser pulse energy, the spot size and the thickness of titanium in energy absorption in LIFT process was theoretically analyzed with Lambert-Beer and a thermal conductive model. After comprehensive analysis, mechanical damage was found to be the dominant factor affecting the size and proliferation ratio of the isolated cells. An orthogonal experiment was conducted, and the optimal conditions were determined as: laser pulse energy, 9 μJ; spot size, 60 μm; thickness of titanium, 12 nm; working distance, 700 μm;, glycerol, 2% and alginate depth, greater than 1 μm. With these conditions, along with continuous incubation, a single cell could be transferred by the LIFT with one shot, with limited effect on cell size and viability.

**Conclusion:**

LIFT conducted in a closed chamber under optimized condition is a promising method for reliably isolating single cells.

## Background

Single cell technology is used to study the growth, metabolism and apoptosis of individual cells [1]. Recently, it has been widely applied to studies of personalized medicine [2], oncology [3], cardiovascular disease [4], fertility [5] and AIDS [6]. The most crucial step for the implementation of single cell technology is isolation of a single cell.

Currently, there are several methods developed to separate an individual cell, including fluorescence activated cell sorting (FACS), magnetic activated cell sorting (MACS), limiting dilution, micro-chips, laser capture micro-dissection (LCM) and optical tweezers. In FACS or MACS, the cells are labeled with fluorescence activated antigen or protein. The cells are then separated by either fluidic or magnetic force, respectively. However, these methods cannot be used to obtain single cells of rare sample or those lacking a known antigen [7–9]. Limiting dilution is the most used method of single cell separation. The cell suspension is repeatedly dilute into new medium untill the density of the cells is below 10 cells/mL. Although it is simple and easy to use, but the target cell cannot be traced since there is no signal feedback [10], and its randomness limits its applications [11, 12]. With the development of micro-fluidics, various chips have been prototyped to capture single cells basing on the certain properties of the cells, such as size [13, 14], rigidity [15] and metabolic function [16, 17]. However, these chips cannot be used to isolate cells that lack clearly distinguishing characteristics [18]. In LCM, cell samples are fixed on a glass slide with either ethanol or formalin. The laser burns out the neighbor cells but keeps the target ones for further research [19]. Because the cells are fixed, the isolated cells with LCM are not viable and cannot be cloned [20]. Optical tweezers, which were originally developed to manipulate micro particles, have recently been introduced to manipulate single cells from channel to a culture well [21], but the manual process is inefficient and complicated [22].

Laser-induced forward transfer (LIFT) utilizes the laser with high energy to partially evaporate the materials coated on the glass, resulting in high pressure to reject the remaining material to a work piece, namely the receptor [23]. Recently, LIFT has been developed for biological materials [24, 25], and has been successfully used to transfer stem cells in tissue engineering [26], to obtain a vein with fully function [27], and to deposit DNA or protein on bio-chips [28]. In these applications, a random number of cells were transferred within each laser shot since there is no specific requirement for the number of deposited cells.

In this paper, we use LIFT to isolate single Hela cell. To prevent lossing of viability from contamination and drying, a chamber was developed with polydimethylsiloxane (PDMS) to seal LIFT environment and regulate humidity. The impact of critical parameters of the process, including laser pulse energy, spot size, working distance and the thickness of the scarified layer was investigated comprehensive. In addition, glycerol was added to the medium and a layer of alginate is deposited on the receptor to optimize the LIFT process. The modifications successfully protected the cells from damage.

## Methods

### Laser induced forward transfer setup

**Fig. 1:**
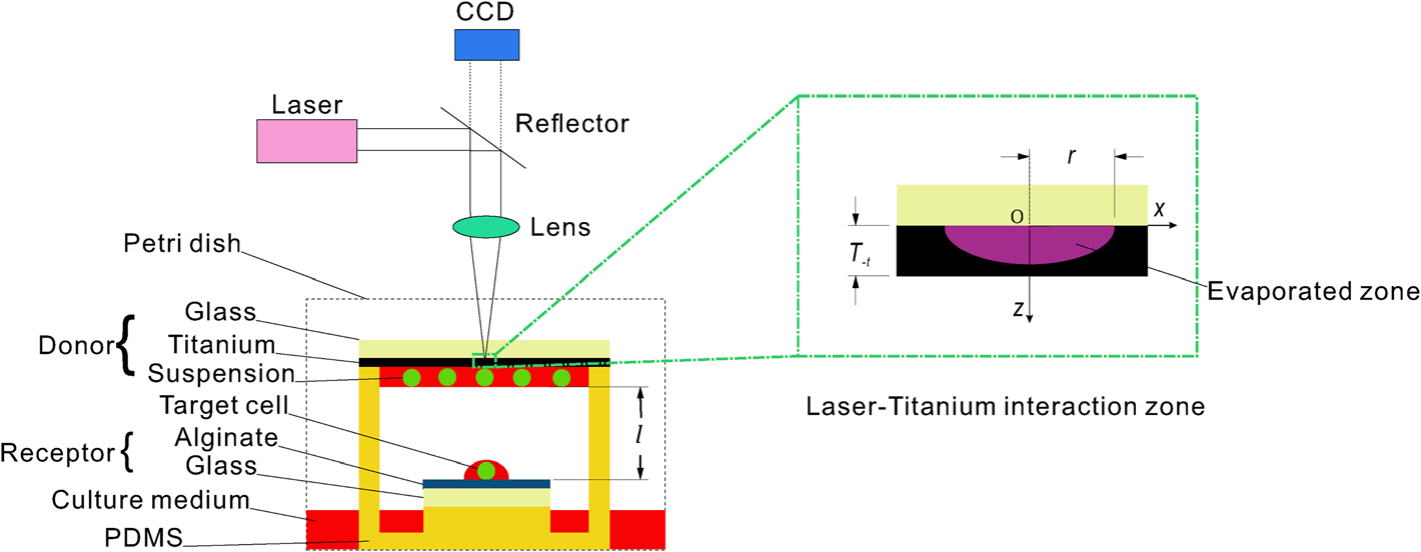
Schematic of single cell transfer with LIFT

As illustrated in Fig. 1, the YAG laser pulse (Dawa 200, Beamtech, China) with pulse width of 5 ns and wavelength of 532 nm passed through a reflector (RBK-25-3-532, Wavelength Opto-electronic, Singapore) and convex lens (LFS-1-150-ET2, Wavelength Opto-electronic, Singapore), and then was focused on the scarificed layer (Titanium) coated on the bottom side of a transparent glass (JGS-1 quartz, In Situ Technology, China). The heat generated by the laser pulse melted the sacrificed layer resulting in shock wave. The target cell in the suspension deposited underneath the sacrificed layer was propelled by the shock wave from the donor (including the glass, sacrificed layer and cell suspension) to the receptor, which was coated with alginate (W201502-Sigma Aldrich, Switzerland). The cells were not able to survive from the process in an open separation environment because of contamination and dry. Therefore, a small chamber made of PDMS was designed to seal the processing environment. The chamber had channels filled with culture medium. And the chamber will located in a culture dish that was filled with culture medium as well. After 2 h of incubation, the humidity inside the chamber reached 100%. Therefore the sealed chamber maintained humidity at the same time as it protect the cells from from contamination and dry.

### Cell preparation

HELA cell (CCL-2™, ATCC, USA) was chosen as the object for its easy-culture, uniformly growth and fast proliferation. Accordingly the cell culture medium is made of 89 vol% DMEM (Dulbecco’s Modified Eagle’s Medium, D5796-Sigma Aldrich, Switzerland), 10 vol% FBS (Fetal Bovine Serum, F6765- Sigma Aldrich, Switzerland) and 1 vol% P/S (Penicillin-Streptomycin, P4333-Sigma Aldrich, Switzerland). The cells were grown at 5% CO_2_, 100% humidity and 37 °C for 5 days untill cells covered over 90% of the flask (3151-Corning, USA). The culture medium was replaced with 4 mL of PBS (Phosphate buffered saline, P5368- Sigma Aldrich, Switzerland) to wash away the dead cells. After dispersing the PBS, 2 mL of trypsin (T4049- Sigma Aldrich) was added to the flask for 3 min to detach the cells. The cells were transferred to a 15 mL tube filled with 8 mL of culture medium and centrifuged. After removing the medium from the tube, fresh medium was added to resuspend cells to a concentration of 10^3^ cells/mL.

### Donor preparation

The base of the donor was quartz glass that was carefully cleaned with Piranha cleaning process. After drying overnight at 80 °C, the sacrificed layer, a layer of Titanium with defined thickness, was deposited on quartz by sputtering coating. The coated quartz was cleaned with ethanol and, rinsed with sterilized water, and then was sterilized with UV light. The prepared cell suspension with cell density of 10^3^ cells/mL was coated on the surface of the Titanium by spin coating process. The depth of the suspension was no more than 30 μm, ensuring a single layer of cells.

### Receptor preparation

Quart was also used as receptor for its great transparency that makes it suitable for microscope. It was sterilized as for the donor, and then Alginate solution (2 wt% in culture medium) was stacked on the quartz by spin coating process.

### Viability analysis of cell after LIFT

The cells were stained with trypanblue (T8154-Sigma Aldrich) to distinguish the dead cells from live ones. The trypan blue was diluted 10 times in PBS, then transferred to the flask of cultured cells. After 30 min, the tryan blue solution was removed and the cell samples were observed under the microscope immediately. The transferred cells were stained after culturing for 1 to 5 days to calculate the proliferation ratio each day.

### Experimental conditions

**Fig. 2:**
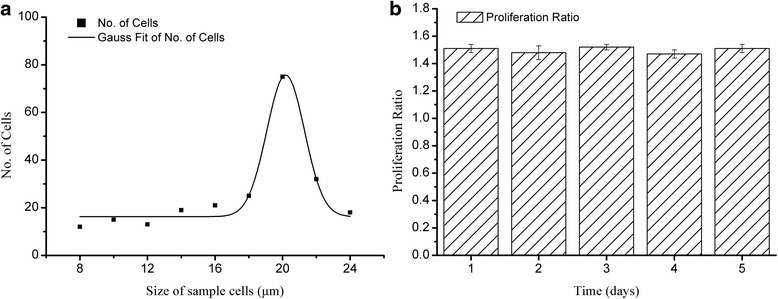
The size distribution and proliferation ration of the untreated cell **a**) the size distribution of the sample cells, **b**) the proliferation ratio of the sample cells

For each of the parameters listed in Table 1, 25 laser pulses were released in order to transfer cells, and all of those cells isolated were cultured immediately in the incubator. The number and the diameter of these cells separated within 25 laser shots were evaluated immediately after the LIFT process to analyze the impact of the key parameters of the process on single cell separation. Ideally, the number should be 25, and the diameter distribution should peak at 20 μm as indicated in Fig. 2 All isolated cells were continuously cultured to verify the viability of cells post LIFT process. The formula for calculating the proliferation ratio was:

**Table 1:** The experimental arrangement of single factor experiments

Parameters	Values
Laser pulse energy (*E*)	1 μJ, 3 μJ, 5 μJ, 7 μJ, 9 μJ, 11 μJ, 13 μJ, 15 μJ
Laser spot size^a^(Diameter, *D*)	20 μm, 25 μm, 30 μm, 35 μm, 40 μm, 45 μm,50 μm, 55 μm, 60 μm, 65 μm
Thickness of Titanium (*T*_*-t*_)	20 nm, 40 nm, 60 nm, 80 nm, 100 nm, 120 nm, 140 nm
Working distance (*l*)	0 μm, 100 μm, 200 μm, 300 μm, 400 μm, 500 μm, 600 μm, 700 μm
Concentration of Glycerol (*d*)	0%, 2%, 4%, 6%, 8%, 10%, 12%, 14%
Thickness of Alginate (*T*_*-A*_)	0 μm, 2 μm, 4 μm, 6 μm, 8 μm, 10 μm

^a^The spot was in reality the functional zone on the surface of titanium, rather than the theoretical spot of the laser. It was altered by varying the focus away from the titanium surface

1 $$ {\eta}_{\Pr oliferation}=\frac{N_i}{N_{i-1}} $$

where *N*_*i*_ indicates the number of the cells in the culture chamber and *i* = 1, 2, 3, 4, 5 is the number of days in culture days, and *N*_0_ is the number of those isolated cells attached to the culture surface in 4 h.

The damage to isolated cells in LIFT process, and their ability to recover were analyzed by comparing their size and proliferation ratio to those of untreated cells (Fig. 2).

## Results

### Effect of laser pulse energy

**Fig. 3:**
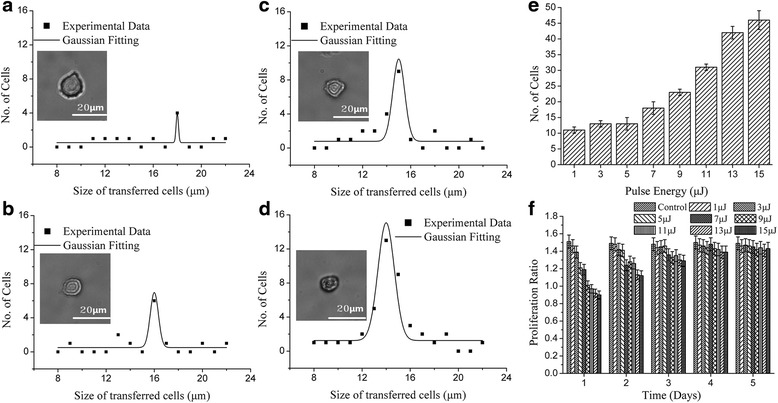
The impact of laser pulse energy. The representative size distribution of the transferred cell at spot size of 40 μm, thickness of Titanium of 60 nm, working distance of 500 μm and no modification in culture medium and receptor with various laser pulse energy of: **a** 1 μJ, **b** 5 μJ, **c** 9 μJ and **d** 13 μJ, **e** the bar diagram of laser pulse energy as function of the number of transferred cells within 25 laser shots and **f** the proliferation ratio change of the of isolated cells with various pulse energy within 5 days

Figure 3a-d presents the cell size distribution when pulse energy was set at 1 μJ, 5 μJ, 9 μJ, and 13 μJ, respectively. After fitting a Gaussian model to the data, the distribution with pulse energy 1 μJ peaked at 18 μm, 5 μJ at 16 μm, 9 μJ at 15 μm, and 13 μJ at 14 μm. For 25 laser shots with 1 μJ, about 10 cells were transferred, resulting in s success rate for single cell isolation of about 40%. When the pulse energy was set at at 9 μJ, 25 cells were separated with success rate of 100%. Above 11 μJ, the number of isolated cell increased above 25 (Fig. 3e). The proliferation ratios of cells are shown in Fig. 3f. Compared with the control cells, all cells isolated by LIFT suffered from a low proliferation ratio on the first day. The proliferation decreased as the pulse energy increased. At 15 μJ the proliferation was less than 1, meaning there were some cell lost the viability. However, the cells recovered their ability to proliferate over time. Only 1 day was needed to recover for samples processed with 1 μJ but samples processed with pulse energy above 11 μJ could not proliferate untill the second day. It was noted that all cell samples had recovered within 4 days. With the target of obtaining a viable single cell, the laser pulse energy should not be set above11μJ.

### Effect of laser spot size

**Fig. 4:**
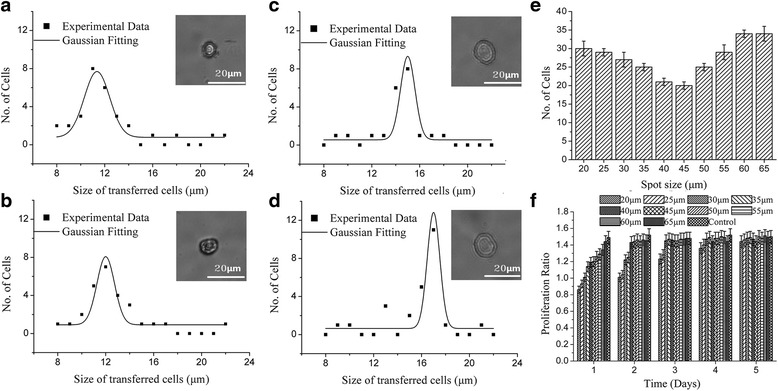
The impact of spot size. The representative size distribution of the transferred cell at laser pulse energy of 7 μJ, thickness of Titanium of 60 nm, working distance of 500 μm and no modification in culture medium and receptor with different laser spot size of: **a** 20 μm, **b** 30 μm, **c** 40 μm and **d** 50 μm, **e** the bar diagram of laser spot size as function of the number of transferred cells within 25 laser shots and **f** the proliferation ratio change of the of isolated cells with various spot size within 5 days

Laser spot size is another important parameter for single cell isolation. Cell size decreased as the spot size was reduced. For instance, Fig. 4a-e presents that samples processed with spot size of 20 μm, 30 μm, 40 μm and 50 μm distributed with peaks at 11 μm, 12 μm, 15 μm and 17 μm, respectively. Meanwhile, the number of the cells isolated from donors decreased from 30 at a spot size of 20 μm to 22 at 40 μm, and then to 21 at 45 μm. As spot size was increased beyond 45 μm, the number of isolated cells increased, 35 at 65 μm as shown in (Fig. 4e). Under these process conditions, the optimal spot sizes were 40 μm and 45 μm, which, the single cell isolation proceeded with 100% success. Furthermore, presented in Fig. 4f), on the first day, the proliferation ratio was less than 1 when the spot size was less than 30 μm The samples transferred at 20 μm or 25 μm spot size started to proliferate on the third day whereas the samples transferred at higher spot size had already fully recovered. The optimal spot size was set between 40 and 45 μm.

### Effect of the thickness of titanium

**Fig. 5:**
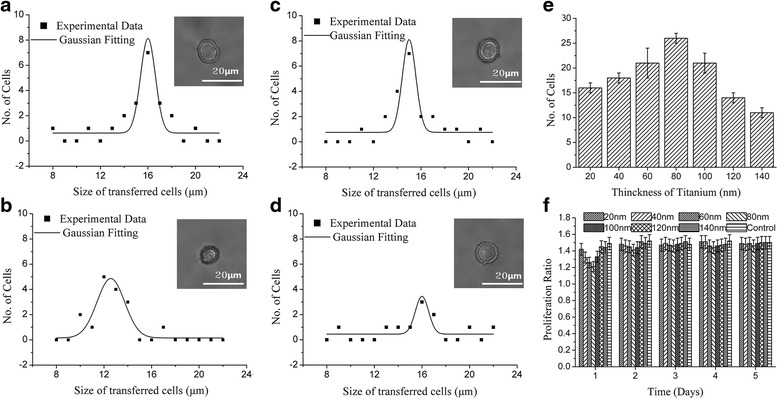
The impact of the thickness of titanium. The representative size distribution of the transferred cell at laser pulse energy of 7 μJ, spot size of 40 μm, working distance of 500 μm and no modification in culture medium and receptor coated with various thick Titanium: **a** 20 nm, **b** 60 nm, **c** 100 nm and **d** 140 nm, **e** the bar diagram of thickness of Titanium as function of the number of transferred cells within 25 laser shots and **f** the proliferation ratio change of the of isolated cells with various thickness of Titanium within 5 days

Next, the effect of titanium thickness on LIFT results was examined. The peaks of the cell-size distribution first deceased from 16 μm at 20 nm to 12 μm at 60 nm of titanium, then rose up to 16 μm at 140 nm, as shown in Fig. 5a-d. The number of cells peaked when the titanium thickness was 80 nm as indicated in Fig. 5e. Although not every laser shot propel a cell successfully, multiple cells were never transferred within one pulse. The proliferation ratio, was the lowest (1.2) 80 nm titanium, and increased at thinner or thicker titanium settings as shown in Fig. 5f. After 2 days post-transfer culture, the cells had recovered. 80 nm titanium thickness resulted in lowest day-1 proliferation ratio, but a high rate of success for separating individual cells with a single laser shot. Therefore, 80 nm is the optimized thickness for titanium this process.

### Effect of working distance

**Fig. 6:**
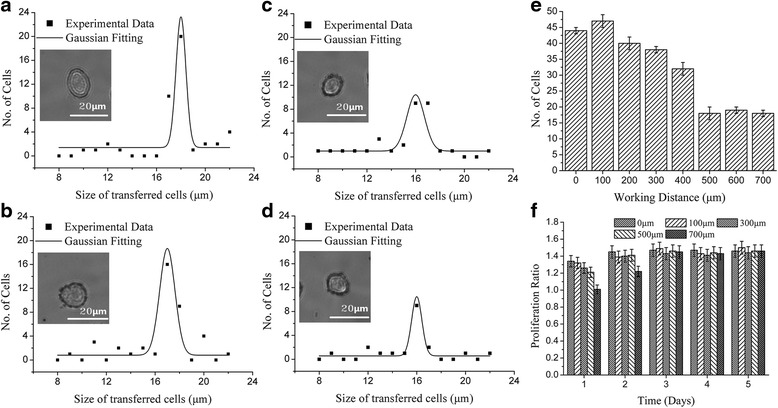
The impact of working distance. The representative size distribution of the transferred cell at laser pulse energy of 7 μJ, spot size of 40 μm, thickness of Titanium of 60 nm and no modification in culture medium and receptor with different working distance of : **a** 0 μm, **b** 100 μm, **c** 300 μm and **d** 500 μm, **e** the bar diagram of working distance as function of the number of transferred cells within 25 laser shots and **f** the proliferation ratio change of the of isolated cells with various working distance within 5 days

We next investigate the working distance, the gap between donor and receptor. As the working distance was varied, the peak cell-size distribution after LIFT changed from 18 μm to 16 μm, as illustrated in Fig. 6a-d but there were 47 cells propelled with a working distance of 100 μm; However, as the working distance inceased above 100 μm, there was a dramatic drop to 20 cells at 500 μm as shown in Fig. 6e). Once the working distance was set to over 500 μm, the number of cells separated was stabilized around 20. After 1 day in culture, the proliferation ratio varied from 1.01 to 1.34. However, after 2 days in culture the cells seemed to recover from the damage. To optimize cell number, size, and proliferation, the working distance should be set in the range of 500 to 600 μm.

### Effect of concentration of glycerol

**Fig. 7:**
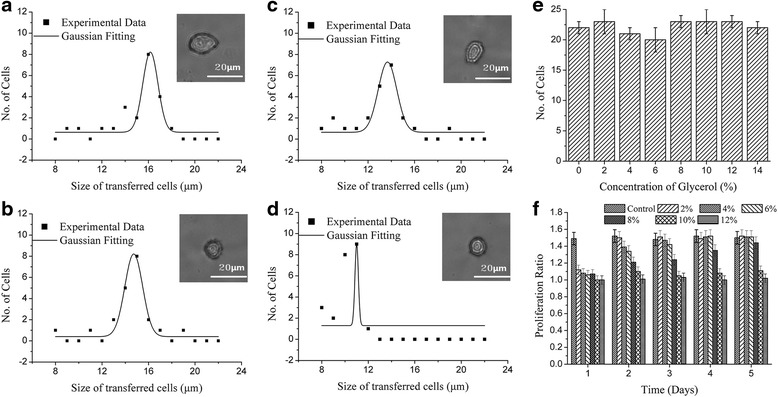
The impact of glycerol concentration. The representative size distribution of the transferred cell at laser pulse energy of 7 μJ, spot size of 40 μm, thickness of Titanium of 60 nm working distance of 500 μm and no modification in receptor but adding to culture medium with different concentration of glycerol: **a** control (0%), **b** 4%, **c** 8% and **d** 12%, **e** the bar diagram of glycerol concentration as function of the number of transferred cells within 25 laser shots and **f** the proliferation ratio change of the of isolated cells with various glycerol concentration within 5 days incubating with no modified culture medium

To optimize droplet formation, glycerol was added into culture medium to increase the viscosity of the medium. Figure 6a-d shows that cell size shrank from 16 μm for medium lacking glycerol to 15 μm, 14 μm, then to 12 μm for those isolated in medium with 4, 8 and 12% glycerol, respectively. Glycerol had no effect on the number of cell separated as illustrated in Fig. 7e. In contrast, glycerol dramatically influenced cell proliferation. Figure 7f showed that the cells did not grow on day-1 after LIFT isolation process with glycerol and lower concentration of glycerol, cells recovered the ability to proliferate. But the cells processed with higher concentration of glycerol took more time to recover. Cells transferred with 10 and 12% glycerol in culture medium survived the process but did not recover the ability to proliferation after 5 days. Therefore, the glycerol concentration should not be above 10% and it should be ideally set between 2–4%.

### Effect of alginate

**Fig. 8:**
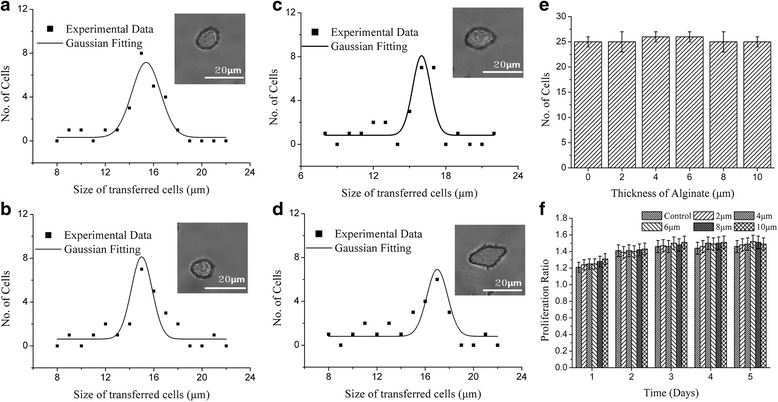
The impact of Alginate. The representative size distribution of the transferred cell at laser pulse energy of 7 μJ, spot size of 40 μm, thickness of Titanium of 60 nm working distance of 500 μm, no modification in culture medium but alginate layer coated on the receptor with different height: **a** control (0 μm), **b** 2 μm, **c** 6 μm and **d** 10 μm, **e** the bar diagram of thickness of alginate as function of the number of transferred cells within 25 laser shots and **f** the proliferation ratio change of the of isolated cells with various thickness of alginate within 5 days

Alginate (2% w/v) was used to form a soft layer on the receptor to ensure that the isolated cell had a soft landing. The peak cell-size shifted from 14 μm to 17 μm as the thickness of alginate increase from 0 μm (control) to 10 μm (Fig. 8a-d). Alginate thickness did not affect the number of isolated cells as expressed in Fig. 8e. But Fig. 8f indicated that alginate did appear to increase the proliferation ratio slightly, in a dose-dependent manner. However, there was minimal increase in the proliferation ratio between 2 μm and 10 μm alginate. Therefore, we concluded that 2 μm of alginate is sufficient to protect the cells from damage.

### Orthogonal optimization

To optimize LIFT single cell isolation, we measured cell number, peak size and proliferation ratio in an orthogonal experiment including three-levels and four-factors (Table 2). As in the single factor experiment, 25 laser pulses were used to propel cells. All cells were counted and measured. Cell size were graphed and fitted with a Gaussian curve to obtain the peak cell diameter. The proliferation ratios were calculated after 3 days of culturing post transfer. The experiment was conducted three times for each condition.

**Table 2:** The result of orthogonal experiment

No.	*E* /μJ	*D* /μm	*T*_*-t*_/nm	*l*/μm	No. of Cells			Peak Diameter^a^			Proliferation Ratio^b^		
1	5	45	80	500	24	25	24	16	16	17	1.47	1.48	1.46
2	5	30	40	300	21	22	22	15	14	14	1.3	1.32	1.31
3	9	60	40	500	18	16	19	13	13	13	1.33	1.33	1.32
4	9	30	80	700	35	37	37	15	13	15	1.35	1.32	1.36
5	5	60	120	700	16	14	14	19	18	19	1.52	1.54	1.49
6	9	45	120	300	24	25	24	15	16	16	1.41	1.46	1.44
7	13	45	40	700	31	29	33	11	12	12	1.12	1.09	1.07
8	13	60	80	300	47	46	38	13	13	14	1.26	1.18	1.22
9	13	30	120	500	42	38	40	12	12	11	1.13	1.11	1.07
Mean I	20.223	32.667	23.447	19.777	No. of Cells								
Mean II	26.110	26.553	24.663	27.333									
Mean III	28.110	15.223	26.333	27.333									
Range	7.887	17.444	2.886	7.556									
Mean I	16.443	13.443	13.000	14.443	Peak Diameter								
Mean II	14.333	14.557	14.663	13.667									
Mean III	12.223	15.000	15.337	14.890									
Range	4.200	1.557	2.337	1.223									
Mean I	1.433	1.290	1.243	1.323	Proliferation Ratio								
Mean II	1.370	1.333	1.343	1.340									
Mean III	1.177	1.357	1.393	1.317									
Range	0.256	0.067	0.150	0.023									

^a^The size distribution of transferred cell was fitting with Gaussian curve, and the peak size was defined as peak diameter with unit of μm

^b^The proliferation ratio here was the proliferation ration after 2 days post-transfer culturing with the consideration of cell’s self-recoverability

**Fig. 9:**
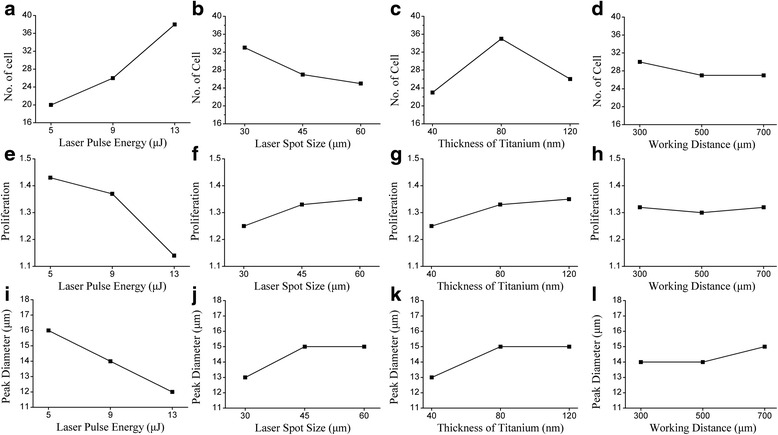
The effects of main parameters, the influence of laser pulse energy, laser spot size, thickness of titianium and working distance on the number of cells presented in **a**-**d** respectively, their impact on the proliferation ratio presented in **e**-**h** respectively, and their impact on the peak diameter of transferred cells presented in **i**-**l** respectively

Figure 9 summarizes the results of this orthogonal experiment. The number of cells increased as pulse energy increased, spot size decreased or working distance decreased. The number of cells peaked as titanium thickness neared 80 nm. The peak diameter of the separated cells, as well as their proliferation ratio, increased as the pulse energy decreases and as the spot size, titanium thickness or working distance increased. Because the goal was to isolate an individual cell with maximum size and maximum proliferation ratio, the optimal LIFT process parameters should be laser pulse energy, 9 μJ; spot size, 60 μm; thickness of titanium, 12 nm; working distance, 700 μm. Spot size is the most critical factor for cell number, with a range of 17.444, followed by pulse energy (range = 7.887), thickness of titanium (range = 2.886) and working distance (range = 7.556). Pulse energy is the predominant contributor to the peak diameter with a range of 4.200, followed by thickness of titanium (range = 2.337), spot size (range = 1.557) and working distance (range = 1.223). For proliferation ratio, pulse energy had the largest effect (range = 0.256), followed by thickness of titanium (range = 0.150), spot size (range = 0.067) and working distance (range = 0.023).

## Discussion

### Mechanism of single cell isolation

For the LIFT process, the only energy source is the laser pulse. The laser interacts with the sacrificed layer (as shown in Fig. 1, the laser-titanium interaction zone), which transforms the energy of laser to thermal effect leading to evaporation of Titanium. Based on thermal conductive theory, the critical depth of melted titanium could be calculated as

2 $$ D=\frac{I_m\left(z,t\right)}{\pi {r}^2\rho \left({C}_p\varDelta T+{L}_f+m\hbox{'}{L}_v\right)} $$

where *I*_*m*_(*z*, *t*) indicates the laser energy absorbed by titanium, *z* the position along the depth direction, *t* the time, *r* the radius of laser spot size, *ρ* the density of titanium, *C*_*p*_ the specific heat capacity of titanium, *ΔT* the boiling point of titanium, *L*_*f*_ fusion heat, *m* ' evaporation ratio, and *L*_*v*_ the evaporation heat.

According to Lambert-Beer [29], the transformed energy can be described as following

3 $$ {I}_m\left(z,t\right)=\alpha k\left(1-R\right){I}_l\left(x,t\right) \exp \left(-\alpha z\right) $$

where *α* is the absorptance, *k* the transmission efficiency, *R* the reflectivity, and the laser used in the process was an Gaussian spot, so the laser intensity distribution could be described as *I*_*l*_(*x*, *t*),

4 $$ {I}_l\left(x,t\right)={I}_0\cdot \exp \left(-\frac{x^2}{r^2}\right)\cdot \exp \left(-\frac{3.5{\left(t-\tau \right)}^2}{\tau^2}\right) $$

where *x* depicts the position in radius direction, *I*_0_ the amplitude of intensity, *τ* the pulse width of laser.

**Fig. 10:**
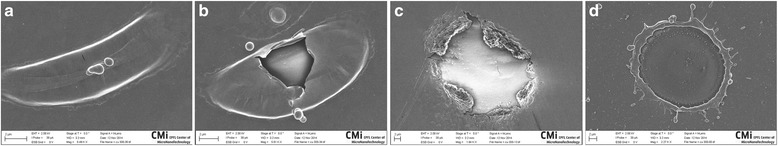
The morphologies of titanium layer after LIFT process, **a** a bump under pulse energy of 2 μJ, spot size of 45 μm, titanium with thickness of 160 nm, **b** a broken bump under pulse energy of 2 μJ, spot size of 45 μm, titanium with thickness of 100 nm, **c** a spot with shrank edge under pulse energy of 2 μJ, spot size of 45 μm, titanium with thickness of 80 nm, **d** a spot completely ablated under pulse energy of 2 μJ, spot size of 45 μm, titanium with thickness of 40 nm

From Eq. (2), the depth of ablated titanium significantly depends on the laser fluency as well as the thermal properties of titanium. Depending on laser, titanium within the critical depth would be evaporated to generate the cavitation. Because of differences in critical depth and the thickness of Titanium, there were four types of morphologies observed on the titanium after LIFT: bump, broken bump, spot with shrunken edge and spot completely ablated as shown in Fig. 10. The four different morphologies mainly resulted from the hybrid functions of high pressure and the constrain of titanium itself. At a given laser fluency, the thicker the titanium results in stronger constrain is, and the morphology changes from a spot completely ablated to a spot with shrank edge, then to a and lastly to a bump. As seen in Eqs. (3) and (4), increasing pulse energy and decreasing the spot size increase laser fluency.

**Fig. 11:**
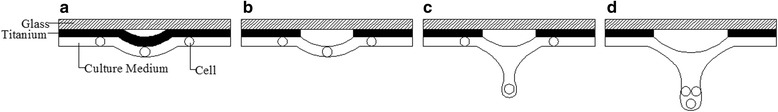
The types of jet formation, **a** bump with titanium partially ablated, **b** bump with titanium completely ablated, **c** narrow jet with an individual target cell in the consequent droplet, **d** less control jet with an multiple cells in the consequent droplet

**Fig. 12:**
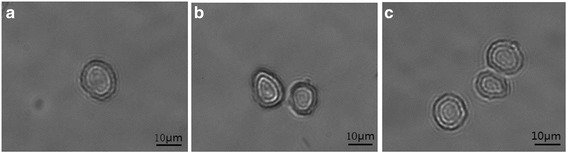
Cell (s) transferred with one laser pulse, **a** an individual cell transferred with a bump jet or narrow jet, **b** two cells transferred with wild jet generated by pulse energy of 4 μJ, **c** three cells transferred with a wild jet generated by pulse energy of 8 μJ

The cavitation resulting from the ablation of titanium expanded with the energy converting to deformation of the sacrificed layer if any, viscous dissipation energy, surface energy, and potentially the kinetic energies to forming jets root from Rayleigh or Plateau-Rayleigh instability [30]. In Newtonian fluids, the jettability significantly depends on the Ohnesorge number *Oh* = *η*/(*ρ*_*l*_ *σR*)^1/2^ and Weber number *We* = *ρ*_*l*_ *RU* ^2^/*σ* where *η* is the zero-shear viscosity, *σ* is the surface tension, *R* is the characteristic length that could be considered as the radius of the laser spot, and *ρ*_*l*_ is the density of medium. Increasing the *Oh* number, which mainly dependes on the property of the medium, helps to constrain the titanium deformation and suppress the jet formation. *We* number is influenced by velocity and medium. By varying the *Oh* number and the *We* number, the jet behavior changes from a bump with titanium partially ablated, to a bump with titanium completely ablated, to a well defined jet, then to a less control one as explained in Fig. 11. In consequence, a single target cell, may either not be transferred, may be isolated precisely, or may be separated along with other cells within one laser pulse, as presented in Fig. 12.

**Fig. 13:**
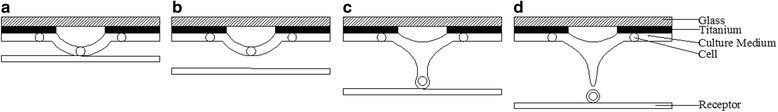
The mechanism of working distance affecting isolation. **a** bump within contact to receptor, **b** bump without contact to receptor, **c** jet without droplet, **d** jet within droplet

The working distance had a limited effect on jet formation but it influenced the process as presented in Fig. 13. With the receptor close to the donor, cells could be transferred to the receptor even when there was not jet formed, as in Fig. 10a-b. But in this case, the number of separated cells was unpredictable because the cells were separated by the capillary force. However, once the working distance was equal to, or larger than the length of jet generated by cavitation, the cells could be precisely transferred.

When the laser energy was raised from 1 μJ to 15 μJ, the laser fluency increased from 79.58 mJ/cm^2^ to 1193.69 mJ/cm^2^. And the jet formation changed through four types, a bump with titanium partially ablated, a bump with titanium completely melted, a narrow jet, and a less controlled jet. Accordingly, the number of cell rose up from 11 to 47.

Conversely, in Fig. 4, the spot size was enlarged from 20 to 65 μm, the laser fluency decreased from 557.06 mJ/cm^2^ to 52.74 mJ/cm^2^, causing the jet to narrow down and even disappear, so there were fewer cells isolated successfully. The spot size also changed the *Oh* number and *We* number by shifting the value of *R*. With a larger spot, a less control jet could form, with which multiple cells were transferred. Generally, increasing the radius of spot size decreased the number of cells separated with 25 laser shots but eventually the number increased again.

The function of the titanium was to convert laser energy to drive the cell separation. But in LIFT process, the thickness of titanium should be carefully selected. On one hand, Titanium functionally absorbs the laser energy to induce cavitation. On the other hand, if not completely evaporated by laser, the remained titanium constrains the expansion of the cavitation and consums the energy to deform. When the titanium is thin (20 nm), The LIFT process is not efficient enough to to isolate cell successfully. In contrast, when the titanium is thick (140 nm), the laser fluency was fully transformed into the process which was 139.26 mJ/cm^2^, but some of the energy was wasted in deformation as shown in Fig. 10a and b).

The critical distance within which the jet was stably formed was 500 μm, so the working distance should be set at least 500 μm to ensure that only a target cell is isolated with each laser pulse.

Modifying the culture medium with glycerol mainly varied the properties of the medium, especially the viscosity. As the glycerol concentration increased, more energy were consumed by viscous dissipation during jetting. As a result, adding glycerol increased the chance that a cell was not transferred with each shot.

### The cell damage

The cells suffered from three types of damage during the LIFT isolation process : mechanical, thermal and UV light. The damage can be recognized by a reduced cell radius or a reduced the proliferation ratio.

The mechanical damage results from three processes. During cavitation bubble expansion, the isolated cell was subject to the high pressure. Duringthe jetting process, the cell is rapidly accelerated to a high velocityThen the cell decelerates when it lands on the receptor. Young reported that the velocity can be high as 1000 m/s and the acceleration/deceleration can be 10^5^-10^9^ g [31, 32]. The pressure, acceleration and deceleration lead to high shear stress on the cell, which shrinks the cell and slows the proliferation. Since the pressure is generated from energy absorbed by titanium, the laser fluency, the thickness of titanium and the working distance are the main factors affecting the shear stress on the cell.

In addition, the interaction between the laser and the titanium produces heat. The heat injures cells by deactivating the enzymes, denaturing protein and carbonization [33]. In fact, the thermal damage is dependent on both temperature and exposure time. Within several nanoseconds, the thermal effect zone was found to be dominated by Fourier heat conduction meaning that the zone was confined to within several micrometers depth. With a 30 μm thick suspension, the thermal injury of separated cell was negligible in this study.

UV lights can kill cells effectively, but in LIFT with a sacrificed layer, the UV light was constrained to the interface between the glass and titanium. Even with a 20 nm thick layer of titanium will absorbed, only 40% of laser passed through the sacrificed layer. Using Lambert-Beer law, with less than 4 μJ of pulse energy and 45 μm of spot size, 60% of laser radiation was absorbed by titanium, even with only a 20 nm thick layer. For the other 40% of laser fluency, the damage threshold depth in culture medium was estimated to be about 2 μm meaning that only 7% of the suspension was affected by UV light, resulting in minimal injury. So in this paper, UV light damage was negligible also.

Among mechanical, thermal and UV damage, under the conditions used in our experiments, mechanical stress was the dominant factor contributing to cell damage. Since the pressure resulted from energy absorbed by titanium, the laser fluency and the thickness of titanium were the main factors affecting the shear stress acting on the cell. As shown in Figs. 3 and 4, increasing the laser pulse energy, reducing the laser spot diameter helped to reduce the cell size, as well as to slow down the proliferation of the transferred target cell. Increasing Titanium thickness from 20 nm to 80 nm decreased the cell size and the proliferation because the titanium contributes on the conversion of energy to cavitation. When it was greater 80 nm, the cells shrank less and the proliferation ratio increased. As previously mentioned, the working distance had a limited impact on jetting, but decreasing the length of path that the isolated cell had to pass through decreased the mechanical damage to the cell, leading to larger cells with higher viability., Glycerol is used to increase the viscosity to form a better droplet but itchanged the osmotic pressure of the medium, making the cell smaller, and reducing the viability. Coating alginate on the receptor was a method to provide enough cushion to reduce the deceleration during the landing process.

## Conclusions

We have analyzed the effects of laser pulse energy, laser spot size, thickness of Titanium, working distance, glycerol and alginate on the number, size and proliferation ratio of cells isolated with LIFT. It revealed that the laser fluency and the thickness of titanium were the main factors affecting the viability of isolated cell because they influence the energy introduced into the process. Providing a sufficient work distances and increasing the viscosity with glycerol helped to control the cell transfer and coating with alginate was employed to soften the cell landing.

The optimal settings for obtaining a viable cellare: laser pulse energy, 9 μJ; spot size, 60 μm; thickness of titanium, 12 nm; working distance, 700 μm, 2–4% glycerol in culture medium and alginate thickness greater than 1 μm.

## Abbreviations

DMEM: Dulbecco’s modified eagle’s medium
FACS: Fluorescence activate cell sorting
FBS: Fetal bovine serum
LCM: Laser capture micro-dissection
LIFT: Laser induced forward transfer
MACS: Magnetic activate cell sorting
P/S: Penicillin-streptomycin
PBS: Phosphate buffered saline
PDMS: Polydimethylsiloxane
